# Patients’ Knowledge About the Endoscopic Ultrasound-Guided Fine Needle Aspiration of Pancreas Is Not Enough

**DOI:** 10.5152/tjg.2023.22380

**Published:** 2023-06-01

**Authors:** Linxia Shen, Jianying Lu, Ying Gu, Wenjuan Shen, Junfang Qi

**Affiliations:** First Affiliated Hospital of Soochow University, Suzhou, China

**Keywords:** Pancreatic lesions, endoscopic ultrasound-guided fine needle aspiration biopsy (EUS-FNA), pancreatic cancer

## Abstract

**Background:**

The aim of the study is to evaluate the extent to which patients acquired necessary knowledge about pancreatic endoscopic ultrasound-guided fine needle aspiration and assess what should be more focused on in the informed consent process.

**Methods:**

Adult patients enrolled in this study had pancreatic lesions confirmed by regular imaging and planned to undergo the first pancreatic endoscopic ultrasound-guided fine needle aspiration. These patients were asked to complete a questionnaire, including indications, possible results, downstream events, the risk for false-negative and malignant lesions, and so on. Then we conducted a long-term follow-up of these patients to obtain the final results.

**Results:**

Most people (94.25%) correctly recognized that the indication of pancreatic endoscopic ultrasound-guided fine needle aspiration was to exclude malignant lesions. Almost all patients knew that the results could be benign or malignant, while the number of people who were aware of non-diagnostic (22%), indeterminate (18%) outcomes, and the possibility of further testing (20%) after the endoscopic ultrasound-guided fine needle aspiration has decreased significantly. Finally, we got that the false-negative rate and percentages of malignancy were 17.81% and 83.91%, while 98% of participants did not recognize that there is a false-negative risk of endoscopic ultrasound-guided fine needle aspiration and more than 2/3 of participants did not know how much risk they might have for malignant lesions.

**Conclusions:**

A high proportion of patients who received endoscopic ultrasound-guided fine needle aspiration could identify the indication for this procedure but remained unaware of possible outcomes, downstream events, especially the risk for false-negative and malignant lesions. It is necessary to improve the quality of dialogue between clinicians and patients, and the information about the risk of false-negative and malignancy may need to be emphasized in the informed consent process.

**Main Points**

- The present study aimed to assess the extent to which patients acquired necessary knowledge about endoscopic ultrasound-guided fine needle aspiration (EUS-FNA) during the informed consent process.
- A high proportion of patients who received EUS-FNA could identify the indication for this procedure but remained unaware of possible outcomes, downstream events, especially the risk for false-negative and malignant lesions.
- Based on our results, we believe that it is necessary to improve the quality of dialogue between clinicians and patients during the informed consent process.

## INTRODUCTION

Endoscopic ultrasound-guided fine needle aspiration (EUS-FNA), a technique that punctures the target tissue with a special puncture needle through the endoscopic tube under the guidance of endoscopic ultrasonography to obtain a histological cytological diagnosis, was developed in 1990, and its application in the diagnosis of pancreatic lesions was first reported by Vilmann in 1992.^1^ In recent decades, it is used widely and plays an important role in the diagnosis of pancreatic lesions.^2-4^

Pancreatic cancer is one of the most fatal cancers in the world due to the limited early detection and diagnosis techniques, so most patients are always found to be in the advanced stage of pancreatic cancer and have distant metastasis when they see a doctor, which often indicates a poor prognosis.^5^ Therefore, it is of great significance for patients to make a clear diagnosis in the early stage and guide the formulation of medical strategies. For the diagnosis of pancreatic lesions, there are traditional methods, such as computed tomography (CT)-guided biopsy and transabdominal ultrasound-guided biopsy. However, with the development and application of endoscopic ultrasonography, several studies in recent decades have pointed out that pancreatic EUS-FNA has a higher detection rate and accuracy than those of the 2 former techniques^6-8^ and has the advantages of shorter puncture distance and higher safety,^9-13^ which plays an indispensable role in the evaluation of pancreatic lesions. The different results of pancreatic EUS-FNA including benign and malignant, non-diagnostic and indeterminate indicate different risks of malignancy and different medical strategies next. For example, a benign result suggests that a lesion is relatively unlikely to be a malignant tumor, requiring no or only limited follow-up, while patients with non-diagnostic or indeterminate results will need further examination (e.g., repeated EUS-FNA, positron emission tomography-computedtomography (PET-CT), and pathological specimens plus more items). Even in some cases where the diagnosis cannot be confirmed, laparotomy may be required to perform diagnostic resection of pancreatic lesions to obtain surgical and pathological results to assist in diagnosis.^14^

Knowing the relevant knowledge about EUS-FNA and understanding its possible risks and benefits is particularly important for patient decision-making. In the routine medical procedure, the clinicians will communicate with the patients before the operation to inform them of the knowledge including indications, possible results, possible complications, downstream events, risk of malignant lesions, and so on. Patients’ acquisition of pancreatic EUS-FNA knowledge mainly comes from preoperative communication and informed consent form, but considering unequal medical background knowledge, limited explanation tools, and different levels of education received by patients, we speculate that different patients may have different degrees of knowledge about EUS-FNA acquisition. Therefore, the main purpose of this study was to evaluate the extent to which patients acquire relevant knowledge when they agree to receive pancreatic EUS-FNA.

We hypothesized that patients obtained the necessary knowledge about pancreatic EUS-FNA through the informed consent procedure. To this end, we conducted this study on patients who would undergo pancreatic EUS-FNA in the Endoscopy Center of the First Affiliated Hospital of Soochow University.

## MATERIALS AND METHODS

### Study Design and Participants

The study included patients who were admitted to the First Affiliated Hospital of Soochow University from January 1, 2017, to December 31, 2021. Patients who had pancreatic lesions confirmed by routine imaging (such as CT/transabdominal ultrasound/magnetic resonance imaging) and planned to undergo the first EUS-FNA were enrolled in this study. They were asked to complete a questionnaire survey that included 6 questions about pancreatic EUS-FNA and 1 question about education level in 30 minutes after preoperative communication and informed consent was obtained. These questions are described in easy-to-understand words as much as possible, making them easier for patients to understand.

As part of routine medical care, a group of 3 experienced gastroenterologists with more than 5 years of clinical professional experience and an experienced nurse in the Endoscopy Center counseled the patients before the operation to inform them of the knowledge of pancreatic EUS-FNA, including indications, potential outcomes, downstream events, the risk for malignant lesions, and so on, and obtained an informed consent form signed by the patients. For patients who consented to participate in the survey, we collected demographic information and medical history from their medical records, while we withdrew the blank questionnaire from patients unwilling to participate. We finally assessed the patients’ knowledge about the relevant information of the pancreatic EUS-FNA based on the answers given by individual patients in the survey.

In addition, we collected the EUS-FNA pathological results and followed up with the patients to obtain the surgical pathological results, follow-up diagnosis, and their treatment. The pathological report of EUS-FNA is usually reported as one of the following categories: malignant, suspicious for malignancy, atypical cells indeterminate for malignancy, and inadequate. We stipulated that the positive result was a cytology report of malignant or suspicious for malignancy, and the negative result was a cytology report of atypical cells or normal cells. And the final diagnosis was given by combining EUS-FNA pathological results, surgical pathological results, and clinical follow-up results. We used the final diagnosis as the gold standard to evaluate the diagnostic value of EUS-FNA and obtain. This study was approved by the Ethics Committee of the First Affiliated Hospital of Soochow University (no: 2020174), and all patients have signed informed consent forms.

### Statistical Analysis

Qualitative and quantitative variables were analyzed by calculating the mean (standard deviation, SD), frequency, and percentages. Final results (combining EUS-FNA pathological results, surgical pathological results with clinical follow-up results to give the final diagnosis) were used to identify true-positive, true-negative, false-positive, and false-negative cases and calculated sensitivity, specificity, and accuracy. Statistical analyses and graphics were performed using STATA 15.

## RESULTS

### Demographic and Clinical Features

A total of 90 people underwent pancreatic EUS-FNA, of which 87 cooperated with the questionnaire, and the response rate was (87/90). Eighty-seven patients were evaluated in the study, including 51 (58.62%) males and 36 (41.38%) females, whose median age was 62.5 years. Most of the patients have a certain degree of reading literacy. Concerning the trigger for the participants to undergo a biopsy, the vast majority of patients indicated that they planned to have a pancreatic EUS-FNA because of the clinical symptoms of discomfort and the need for diagnosis and treatment (Table 1).

### Results of Survey

**Figure 1.**
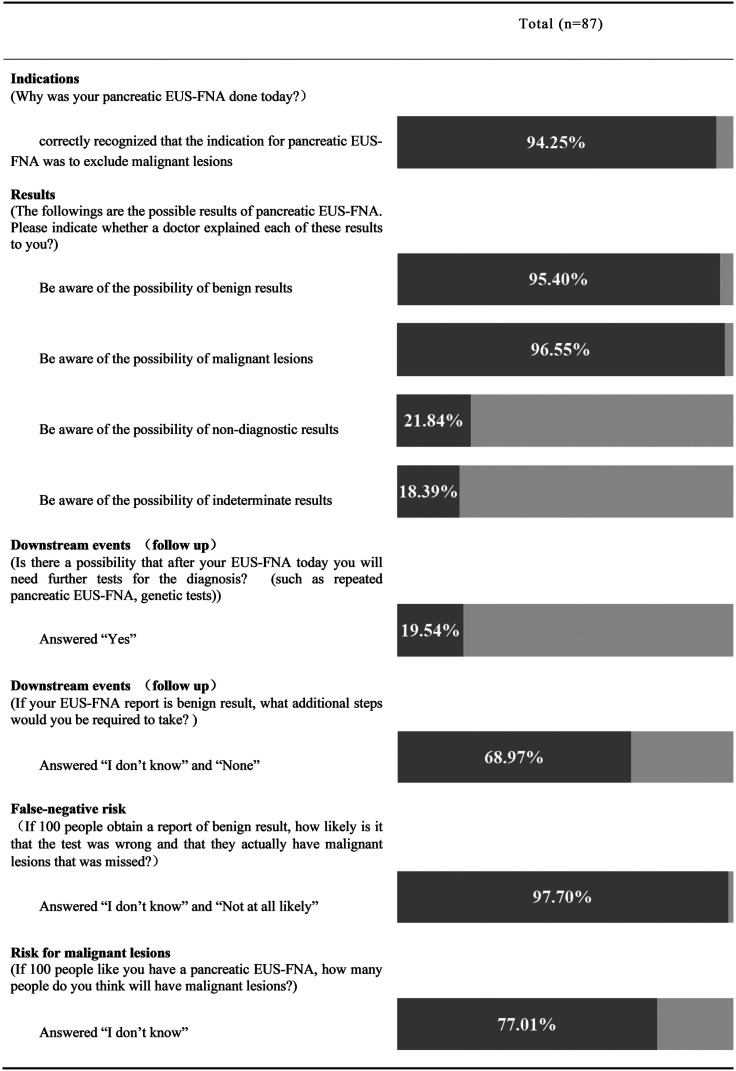
Patients’ knowledge of the indications, results, and follow-up events of pancreatic endoscopic ultrasound-guided fine needle aspiration.

As for the indications for their current EUS-FNA, most patients (94.25%) correctly recognized that the indication for pancreatic EUS-FNA was to exclude malignant lesions (Figure 1).

As for the possible results of EUS-FNA, most patients answered that they knew that the pathological reports of pancreatic EUS-FNA were possibly benign (95.40%) or malignant (96.55%), but fewer patients were aware of non-diagnostic (21.84%) and indeterminate results (18.39%) (Figure 1).

As to whether further tests are needed after the EUS-FNA, only 19.54% of the patients were aware of the possibility of further testing after the EUS-FNA, while 16.09% thought EUS-FNA was the final test and 64.37% said they were unsure (Figure 1).

As for the question of what additional steps patients would be required to take if the EUS-FNA report is a benign result: 68.97% of patients were not aware of the need for follow-up (Figure 1).

**Figure 2.**
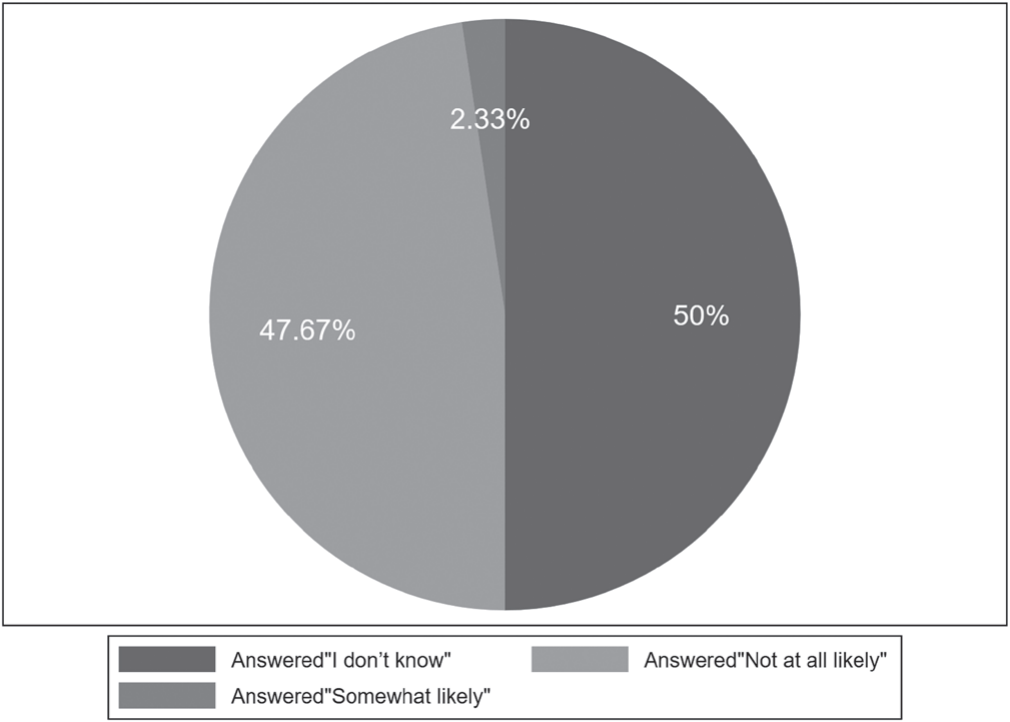
Patients’ answers distribution about false-negative risk.

As for the question of whether there is a false-negative risk of EUS-FNA, 47.67% of patients believed that benign results completely exclude the possibility of malignant lesions and the false-negative risk of benign results was close to 0%, and half of the patients (50%) answered that they did not know how likely the benign results were inaccurate (Figures 1 and 2). Only 2.33% thought that the test had the risk of false-negative results, but the risk was very small, less than 20% (Figure 2).

**Figure 3.**
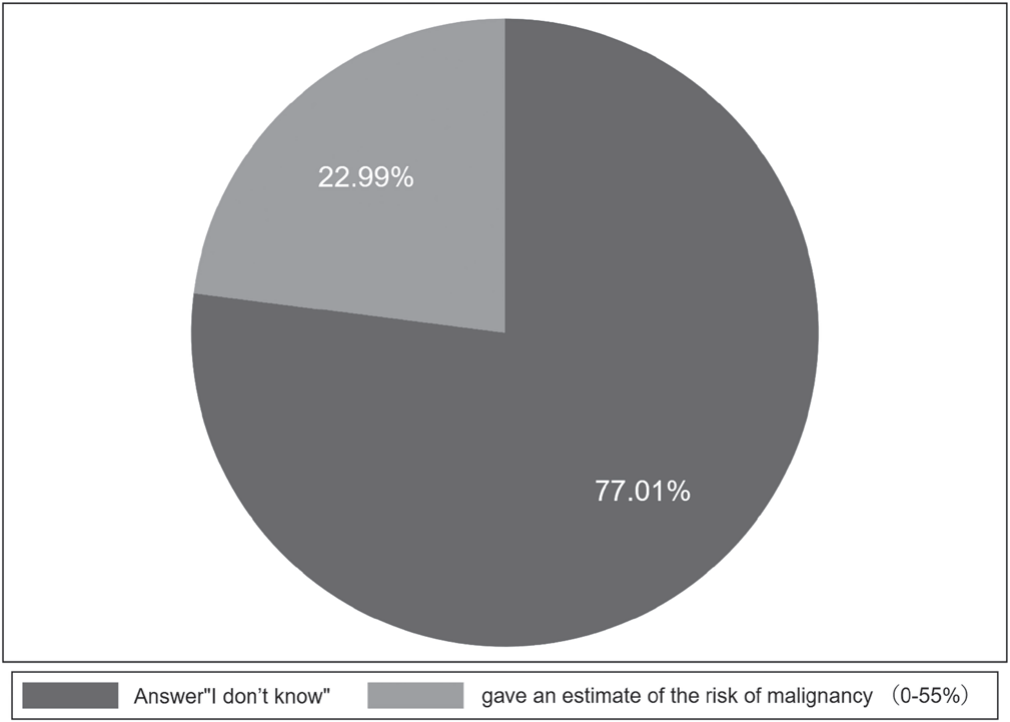
Patients’ answers distribution about risk for malignant lesions.

As for the question of estimating the risk of malignant lesions, more than 2/3 of participants answered that they did not know how much risk they might have for malignant lesions (Figures 1 and 3). Less than 1/3 of patients provided an estimate for the risk of malignant lesions, which ranged from 0% to 55% (Figure 3).

Among the 87 patients enrolled in this study, 57 cases got positive results, 26 cases got negative results, and 4 cases obtained non-diagnostic or indeterminate results. The positive results of 57 cases were confirmed as malignant lesions by postoperative pathology or follow-up, while 26 negative results, of which 13 cases were diagnosed as pancreatic malignant tumors and 13 cases were benign lesions (Table 2). About 4 cases were without clear and definite results by EUS-FNA, of which 3 cases were finally identified as malignant lesions and 1 case was benign.

The sensitivity, specificity, and accuracy of EUS-FNA were 78.08%, 92.86%, and 80.46%, respectively. The false-negative rate of EUS-FNA was 17.81% and the percentages of malignancy were 83.91%.

## DISCUSSION

This study aimed to evaluate patients’ knowledge about pancreatic EUS-FNA by investigating patients who planned to undergo pancreatic EUS-FNA. We found that the knowledge that patients acquired about pancreatic EUS-FNA was obviously less than what the consent form informed them. They had less knowledge about the false-negative rate and lower assessment for the risk of malignant lesions than the reality.

The answers to questions in the survey reflected patients’ knowledge about EUS-FNA to some extent. The survey showed that the vast majority of patients were well aware of pancreatic EUS-FNA indications to rule out malignant lesions and that the results may be benign or malignant, but only a few people knew that the results were possibly nondiagnostic and indeterminate, and further tests might be needed to give the final diagnosis. Some people thought that EUS-FNA was the last test and would give a clear diagnosis. However, the reality was not what they thought it is. In the process of follow-up, not all patients obtained a clear diagnosis through this pancreatic EUS-FNA. In the study, there were indeed 4 cases with nondiagnostic and indeterminate results, of which two patients were diagnosed with pancreatic malignant lesions by further laparotomy and surgical pathological results, one case was confirmed by repeated EUS-FNA, and one case was confirmed by clinical long-term follow-up. Therefore, when informing patients of relevant knowledge and information about EUS-FNA, we might need to emphasize that this test is only an auxiliary method in the process of diagnosis, and there may be uncertain results and the possibility of further tests to strengthen patients’ awareness of EUS-FNA.

In addition, when patients were informed of the results of benign lesions, more than 2/3 of patients did not realize that follow-up was still needed, and only 2.33% were aware of the possibility of false-negative. In the survey, when they were asked, “If 100 people obtain a report of benign result, how likely is it that the test was wrong and that they actually have malignant lesions that were missed,” 2.33% of responders checked the answer “somewhat likely, up to 20 of the 100 people might actually have malignant lesions that were missed,” while the other patients answered, “Not at all likely, a benign result completely excludes the possibility of malignant lesions” or “I don’t know.” Actually, there are 26 people who obtained a report of negative result, and 13 people have malignant lesions that was missed in the research. Indeed, there is always false-negative rate, which can be observed both in our study and in previous research.^15-17^ In the current study, we calculated that the false-negative rate was 17.81%, suggesting that if 100 people with malignant lesions have a pancreatic EUS-FNA, about 17 people may get the wrong result and have malignant lesions that were missed. This gap between patients’ answers and reality seems that the informed consent procedures may leave patients uninformed. In addition, there were studies indicating that FNB has very low false-negative results and its sensitivity is around 92% to 95%, replacing FNA for tissue diagnosis of pancreatic mass lesions.^18^ But the risk of false-negative has not decreased to zero and completely disappeared, which is always observed in any study. We may still need to inform the patient in detail that there is a risk of missed diagnosis of the procedure and what the false-negative rate of the test is according to the survey of the hospital in recent years or guidelines about EUS-FNA.

Furthermore, the current study indicated that patients’ awareness of diseases they may be suffering was poor, and they had a lower assessment for the risk of malignant lesions than the reality. When patients were asked the question about their risk of suffering malignant lesions, 77.01% gave an “I don’t know” answer, and the other patients gave their estimates for the risk of malignant lesions, but the estimates ranged from 0% to 55%. Although previous studies have not clearly indicated the risk of malignant lesions in patients undergoing pancreatic EUS-FNA, it is not difficult to observe that these patients are more likely to have malignant outcomes.^17,19,20^ Because in clinical, considering the combination of medical history and auxiliary examination, doctors often consider the fact that these patients are more likely to have a malignant result if they advise patients to undergo a further pancreatic EUS-FNA test. As the participants in our research, 83.91% of the patients were finally diagnosed with malignant lesions. Previous studies also showed that the proportion of patients with pancreatic EUS-FNA who were finally diagnosed as malignant lesions was not low, basically higher than 70% of malignant outcomes,^6,17,19-22^ much higher than the estimates given by the patients in our study. Therefore, it was observed that although these patients undergoing pancreatic EUS-FNA were more likely to get malignant results, they were not clearly aware of the situation, which suggested that communication about the risk of malignant lesions may need to be emphasized.

Combined with previous research, it can be found that obtaining the necessary knowledge from preoperative communication and informed consent procedure seems to be not only a problem for patients with pancreatic lesions in our research but also in patients with other diseases in previous literature. Several previous studies have also indicated that patients always overestimated the diagnosis and treatment value of a certain medical technology^23^ or the benefits of a certain operation,^24^ which may be due to the unequal knowledge between doctors and patients, the relative professionalism of medical knowledge, and the deviation of focus when patients obtain information.

The current study has some limitations: first, it is a single-center study with small sample size, and more multicenter studies are needed to assess patients’ knowledge of EUS-FNA. Second, patients who did not respond were excluded, and the result may be affected. Third, we did not explore whether patients collected information about pancreatic EUS-FNA from other sources before the procedure, which may cause a certain response bias.

## CONCLUSIONS

A high proportion of patients who received pancreatic EUS-FNA could identify the indication for this procedure but remained unaware of potential outcomes, downstream events, especially the risk for false-negative and pancreatic malignant lesions. So, it is necessary to improve the quality of dialogue between clinicians and patients, and the information about the risk of false-negative and malignancy may need to be emphasized in the informed consent process.

## Figures and Tables

**Table 1.** Demographic and Clinical Features of Study Participants

Demographics	n (%)
Gender, n (%)	
Male	51 (58.62%)
Female	36 (41.38%)
Age (median [IQR], years)	62.5 (13.5)
Education, n (%)	
Less than high school	16 (18.39%)
High school graduate	49 (56.32%)
College graduate	17 (19.54%)
Postgraduate	3 (3.45%)
A medical profession, such as a nurse, etc.	2 (2.30%)
Trigger for EUS-FNA, n (%)	
Incidental imaging detection	6 (6.90%)
Incidental finding on physical examination	0 (0%)
Symptomatic	76 (87.35%)
Others	5 (5.75%)

EUS-FNA, endoscopic ultrasound-guided fine needle aspiration; IQR, interquartile range.

**Table 2.** Pancreatic EUS-FNA Results and Final Results

EUS-FNA Results	Final Results		
	Malignant	Benign	Total
Positive	57	0	57
Negative	13	13	26
Non-diagnostic or indeterminate	3	1	4
Total	73	14	87

EUS-FNA, endoscopic ultrasound-guided fine needle aspiration.

## References

[1] Vilmann P Jacobsen GK Henriksen FW Hancke S . Endoscopic ultrasonography with guided fine needle aspiration biopsy in pancreatic disease. Gastrointest Endosc. 1992;38(2):172 173. (10.1016/s0016-5107(92)70385-x) 1568614

[2] Koul A Baxi AC Shang R et al. The efficacy of rapid on-site evaluation during endoscopic ultrasound-guided fine needle aspiration of pancreatic masses. Gastroenterol Rep (Oxf). 2018;6(1):45 48. (10.1093/gastro/gox017) 29479442 PMC5806417

[3] Collins BT Murad FM Wang JF Bernadt CT . Rapid on-site evaluation for endoscopic ultrasound-guided fine-needle biopsy of the pancreas decreases the incidence of repeat biopsy procedures. Cancer Cytopathol. 2013;121(9):518 524. (10.1002/cncy.21340) 23983161

[4] Iglesias-Garcia J Dominguez-Munoz JE Abdulkader I et al. Influence of on-site cytopathology evaluation on the diagnostic accuracy of endoscopic ultrasound-guided fine needle aspiration (EUS-FNA) of solid pancreatic masses. Am J Gastroenterol. 2011;106(9):1705 1710. (10.1038/ajg.2011.119) 21483464

[5] Mizutani N Mochizuki M Toki M . Assessment of preoperative pancreatic biopsy, cytological/histological review of cell-block-specimens obtained by endoscopic ultrasound-guided fine-needle aspiration: laboratory-based study. Diagn Cytopathol. 2020;48(4):408 413. (10.1002/dc.24358) 31825182 PMC7079018

[6] Saqib M Maruf M Bashir S et al. EUS-FNA, ancillary studies and their clinical utility in patients with mediastinal, pancreatic, and other abdominal lesions. Diagn Cytopathol. 2020;48(11):1058 1066. (10.1002/dc.24523) 32515558

[7] Gonzalo-Marin J Vila JJ Perez-Miranda M . Role of endoscopic ultrasound in the diagnosis of pancreatic cancer. World J Gastrointest Oncol. 2014;6(9):360 368. (10.4251/wjgo.v6.i9.360) 25232461 PMC4163734

[8] Mehmood S Jahan A Loya A Yusuf MA . Onsite cytopathology evaluation and ancillary studies beneficial in EUS-FNA of pancreatic, mediastinal, intra-abdominal, and submucosal lesions. Diagn Cytopathol. 2015;43(4):278 286. (10.1002/dc.23207) 25088987

[9] Cazacu IM Luzuriaga Chavez AAL Saftoiu A Vilmann P Bhutani MS . A quarter century of EUS-FNA: progress, milestones, and future directions. Endosc Ultrasound. 2018;7(3):141 160. (10.4103/eus.eus_19_18) 29941723 PMC6032705

[10] Hewitt MJ McPhail MJW Possamai L Dhar A Vlavianos P Monahan KJ . EUS-guided FNA for diagnosis of solid pancreatic neoplasms: a meta-analysis. Gastrointest Endosc. 2012;75(2):319 331. (10.1016/j.gie.2011.08.049) 22248600

[11] Iglesias-Garcia J Lariño-Noia J Domínguez-Muñoz JE . When to puncture, when not to puncture: pancreatic masses. Endosc Ultrasound. 2014;3(2):91 97. (10.4103/2303-9027.123007) 24955338 PMC4064167

[12] Bournet B Souque A Senesse P et al. Endoscopic ultrasound-guided fine-needle aspiration biopsy coupled with KRAS mutation assay to distinguish pancreatic cancer from pseudotumoral chronic pancreatitis. Endoscopy. 2009;41(6):552 557. (10.1055/s-0029-1214717) 19533561

[13] Napoleon B Alvarez-Sanchez MV Gincoul R et al. Contrast-enhanced harmonic endoscopic ultrasound in solid lesions of the pancreas: results of a pilot study. Endoscopy. 2010;42(7):564 570. (10.1055/s-0030-1255537) 20593334

[14] Ge N Zhang S Jin Z et al. Clinical use of endoscopic ultrasound-guided fine-needle aspiration: guidelines and recommendations from Chinese Society of Digestive Endoscopy. Endosc Ultrasound. 2017;6(2):75 82. (10.4103/eus.eus_20_17) 28440232 PMC5418971

[15] Sugiura R Kuwatani M Yane K et al. Prospective, multicenter, observational study of tissue acquisition through EUS-guided fine-needle biopsy using a 25G Franseen needle. Endosc Ultrasound. 2019;8(5):321 328. (10.4103/eus.eus_66_18) 30880724 PMC6791109

[16] Ding S Lu A Chen X et al. Diagnostic accuracy of endoscopic ultrasound-guided fine-needle aspiration: A single-center analysis. Int J Med Sci. 2020;17(17):2861 2868. (10.7150/ijms.48882) 33162814 PMC7645325

[17] Yoshinaga S Itoi T Yamao K et al. Safety and efficacy of endoscopic ultrasound-guided fine needle aspiration for pancreatic masses: a prospective multicenter study. Dig Endosc. 2020;32(1):114 126. (10.1111/den.13457) 31166046

[18] Bang JY Krall K Jhala N et al. Comparing needles and methods of endoscopic ultrasound-guided fine-needle biopsy to optimize specimen quality and diagnostic accuracy for patients with pancreatic masses in a randomized trial. Clin Gastroenterol Hepatol. 2021;19(4):825-835.e7. (10.1016/j.cgh.2020.06.042) 32652307

[19] Ishikawa T Ohno E Mizutani Y et al. Usefulness of macroscopic on-site evaluation using a stereomicroscope during EUS-FNB for diagnosing solid pancreatic lesions. Can J Gastroenterol Hepatol. 2022;2022:2737578. (10.1155/2022/2737578) 35087769 PMC8789468

[20] Gheorghiu M Sparchez Z Rusu I et al. Direct comparison of ­elastography endoscopic ultrasound fine-needle aspiration and B-mode endoscopic ultrasound fine-needle aspiration in diagnosing solid pancreatic lesions. Int J Environ Res Public Health. 2022;19(3). (10.3390/ijerph19031302) PMC8834989 35162325

[21] Okasha HH Naga MI Esmat S et al. Endoscopic ultrasound-guided fine needle aspiration versus percutaneous ultrasound-guided fine needle aspiration in diagnosis of focal pancreatic masses. Endosc Ultrasound. 2013;2(4):190 193. (10.4103/2303-9027.121239) 24949394 PMC4062270

[22] Banafea O Mghanga FP Zhao J Zhao R Zhu L . Endoscopic ultrasonography with fine-needle aspiration for histological diagnosis of solid pancreatic masses: a meta-analysis of diagnostic accuracy studies. BMC Gastroenterol. 2016;16(1):108. (10.1186/s12876-016-0519-z) PMC5007683 27580856

[23] Singh Ospina N Castaneda-Guarderas A Ward R et al. Patients’ knowledge about the outcomes of thyroid biopsy: a patient survey. Endocrine. 2018;61(3):482 488. (10.1007/s12020-018-1639-8) 29909600

[24] Rothberg MB Sivalingam SK Ashraf J et al. Patients’ and ­cardiologists’ perceptions of the benefits of percutaneous coronary intervention for stable coronary disease. Ann Intern Med. 2010;153(5):307-313. (10.7326/0003-4819-153-5-201009070-00005) 20820040

